# Assessment of Parents’ Preferences for Incentives to Promote Engagement in Family-Based Childhood Obesity Treatment

**DOI:** 10.1001/jamanetworkopen.2019.1490

**Published:** 2019-03-29

**Authors:** Davene R. Wright, Brian E. Saelens, Angela Fontes, Tara A. Lavelle

**Affiliations:** 1 Department of Pediatrics, University of Washington School of Medicine, Seattle; 2 Center for Child Health, Behavior, and Development, Seattle Children’s Research Institute, Seattle, Washington; 3 Department of Psychiatry and Behavioral Sciences, University of Washington School of Medicine, Seattle; 4 NORC at the University of Chicago, Chicago, Illinois; 5 Center for the Evaluation of Value and Risk in Health, Institute for Clinical Research and Health Policy Studies, Tufts Medical Center, Boston, Massachusetts; 6 Tufts University School of Medicine, Boston, Massachusetts

## Abstract

**Importance:**

Family-based treatment (FBT) is recommended for childhood obesity, but even when sought, attrition is high. Extrinsic incentives, which have been effective for improving adult health, could improve FBT engagement.

**Objective:**

To assess parents’ preferences for FBT incentives.

**Design, Setting, and Participants:**

Survey study of parents of children aged 6 to 17 years with obesity (body mass index in ≥95th percentile for age and sex). Parents’ preferences for FBT incentive program attributes were assessed in a discrete choice experiment conducted using a nationally representative, web-based survey in March 2018. Attributes included (1) the monetary value of the incentive, (2) the payment structure, (3) the goal being incentivized, and (4) the person(s) being incentivized. A fractional factorial design was used to create a set of profiles representing hypothetical FBT incentives. Parents were presented with 10 pairs of profiles and asked which would most motivate them in FBT. Parents were also asked about preferences between a small, guaranteed incentive and a lottery for a large incentive. Analyses used a hierarchical Bayesian model and linear regressions.

**Main Outcomes and Measures:**

Parents’ preference for different incentive program attributes and levels.

**Results:**

The nationally representative survey had a 41.4% eligibility rate (n = 339) and a 89.7% completion rate (n = 304). A total of 53.3% of respondents (weighted percentage) were non-Hispanic white, 42.6% had an annual household income less than $50 000, and 28.3% had a bachelor’s degree. Parents preferred higher incentives, although they were willing to accept lower-value incentives if both the parent and the child (vs the child alone) were required to meet the goal (mean difference [MD], −$108; 95% CI, −$132 to −$84), if the incentive used a gain-framed vs loss-framed payment structure (MD, −$72; 95% CI, −$85 to −$59), and if physical activity goals were incentivized over weight loss (MD, −$63; 95% CI, −$82 to −$44) or dietary monitoring (MD, −$5; 95% CI, −$1 to $28). Only 20.6% of parents preferred a lottery over a guaranteed payment. Preferences did not vary among demographic or health subgroups.

**Conclusions and Relevance:**

In this study, parent-stated preferences did not align with FBT best practices or behavioral economic theory. A randomized clinical trial could examine whether aligning incentives with preferences or best practices would maximize FBT engagement and behavior change.

## Key Points

**Question:**

What is the parent-preferred structure of financial incentive programs for promoting engagement in family-based childhood obesity treatment?

**Findings:**

In this survey study of 304 parents of children with obesity, participants chose incentives that offered higher payments but were willing to accept lower amounts if the incentives used positive reinforcement, targeted both parents and children, and rewarded physical activity.

**Meaning:**

Participant preferences conflict with family-based childhood obesity treatment best practices as well as behavioral economic best practices for incentivizing behavior change; if incentives are unacceptable to their target populations, it could hinder program uptake and reduce participant self-efficacy.

## Teaser

This survey study assesses preferences of parents of children with obesity for incentive attributes and levels in family-based treatment programs for childhood obesity.

## Introduction

Family-based treatment (FBT) is a weight management approach for children with obesity involving in-person behavioral counseling for diet and physical activity behavior change with parent-child dyads. Best practices for FBT involve at least 26 hours of in-person counseling during a 6-month period.^1^ In addition to intervention sessions, families participating in FBT are also frequently encouraged to provide children with the opportunity to get more than 60 minutes of moderate to vigorous physical activity daily, reduce screen time, keep a record of all foods the parent and child consume as well as the moderate to vigorous physical activity engaged in, and change the home food environment.^1^ Family-based treatment results in sustained improvements in parent and child body mass index (BMI, calculated as weight in kilograms divided by height in meters squared) and is recommended by expert panels.^1,2^

Given the intervention’s intensity, FBT can be challenging and time-consuming.^3,4,5^ Barriers to FBT engagement include child motivation and high out-of-pocket expenditures.^3,4,5,6,7,8^ Attrition rates from pediatric weight management programs range from 27% to 73%.^4^ The longer families stay in FBT, the more effective it is.^9,10,11^ Strategies to reduce attrition and motivate families within this effective treatment program are needed.^9^

Financial incentive programs have long been used to promote treatment engagement and healthy behaviors.^12,13,14,15,16,17,18,19,20^ By providing people with immediate and consistent gratification for engaging in challenging behaviors that have uncertain and delayed benefits, incentives can keep participants engaged long enough to observe the long-term benefits of behavior change.^21^

The structure of financial incentives can influence health behaviors and outcomes. Possible structures include individual vs group incentives for weight loss,^22^ incentives that use gain-framed payments (ie, earning money when you meet your goal) vs loss-framed payments (ie, money allocated up front is taken away when a goal is not met),^14^ lotteries (the chance to win a large prize if the goal is met but no prize or a small prize if the lottery is not won),^14,15^ incentivizing process measures (eg, physical activity time and attending health classes) vs outcome measures (eg, weight change and blood pressure level),^23^ and bonuses for achieving target outcomes.^18^

However, little is known about how financial incentive structures could improve FBT engagement, including initiating and sustaining healthy weight-related behaviors. Previous work assessing preferences for obesity-related incentive programs has focused on adults.^24,25,26^ However, the value and structure of financial incentives that are effective for improving adult health behaviors may not work within an FBT context. Effective FBT is so intensive and structured that financial incentives for families may need to be higher than for less intense adult obesity–related interventions that have been evaluated in the behavioral economics literature to date.^6,15,20^ Further, using loss-framed or lottery-based financial incentives, which are aligned with behavioral economic theory,^14,15^ may be detrimental for children who are still learning to understand how to process progress and failure in association with their actions.^27^ A financial incentive program that aligns with patient preferences may improve FBT initiation and the sustainability of incentive programs for promoting behavior change. The aim of this study was to assess parents’ relative preferences for the characteristics of financial incentive programs that would motivate families in FBT, including the payment structure and the monetary value of the incentive.

## Methods

### Survey and Participants

We fielded a survey in March 2018 using the AmeriSpeak panel, a standing, probability-based panel designed to be representative of the US population of civilian adults. Details on panel characteristics and recruitment are published elsewhere.^28^ AmeriSpeak randomly sampled English-speaking parents of children aged 6 to 17 years to complete a survey about what might motivate them to participate in a family-based childhood weight management program. Parents completed an online consent process before initiating the survey. Screening questions asked parents to report their child’s (or children’s) age in years, sex assigned at birth, and height and weight to determine study eligibility. Participants had to be the parent of a child aged 6 to 17 years with a BMI in the 95th percentile or above. Parents received an incentive worth $5 for completing the survey. The study was approved by the Seattle Children’s Hospital Institutional Review Board. The study followed the American Association for Public Opinion Research (AAPOR) reporting guideline. The full survey instrument is available in the eAppendix in the Supplement. Study data have been previously published.^29^

### Discrete Choice Experiment

We identified attributes (characteristics) of an FBT incentive from the literature on financial incentives and behavior change; other previously conducted discrete choice experiments (DCEs) that studied weight-related behavior change^24,25,26^; data from 23 semistructured qualitative interviews with parents, some of whom had experience with FBT^6^; and expert opinions obtained from child health researchers and health economists. A 6-month FBT program length was chosen in accordance with US Preventive Services Task Force recommendations.^1^ Four attributes were chosen to reflect the characteristics of the incentive program, not the characteristics of the FBT program. Attribute levels were chosen to represent the range of expected values of each attribute based on the literature and expert opinions, as previously described. The attributes (and levels) included total monetary value of the reward over the 6-month program ($150, $220, $290, $360, $430, and $500), the structure of the payment (gain-framed vs loss-framed payments), the goal being incentivized (dietary monitoring, increasing physical activity, or a change in weight), and who had to meet the goal to earn the reward (child only or parent and child). Cognitive interviews conducted in person with a convenience sample (n = 8) were used to solicit parent feedback on the clarity of the survey instrument; the survey was revised accordingly.

With 4 attributes in each profile and each attribute containing 2 to 6 levels, it would be impractical to show parents potential incentive programs representing every permutation of attribute levels. Therefore, a fractional factorial design was used to create a balanced set of profiles that displayed each attribute level an equal number of times. We created 600 incentive program profiles, paired into 300 choice questions. These questions were divided into 30 blocks with 10 choice questions each. Participants were randomized and received 1 block of 10 choice questions, each comparing 2 possible rewards. In accordance with best practices for DCEs, we sought to administer the survey to a minimum of 300 parents.^30^

**Figure 1.**
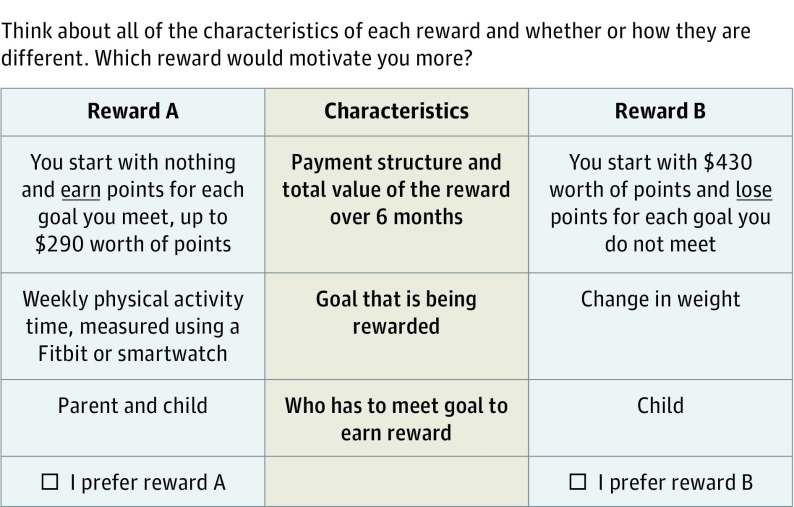
Sample Discrete Choice Experiment Question

The survey included an introduction to the discrete choice scenario (eAppendix in the Supplement). After completing a practice question intended to assess parent comprehension of the DCE task, each parent answered 10 discrete choice questions in which they were asked to choose which of 2 possible incentive program profiles would motivate them more (Figure 1).

A separate survey question was used to assess parent preferences for a small, guaranteed reward or a lottery system for a larger reward with the same mean value. The lottery option was not included as a level in the payment structure attribute to improve the clarity of the survey and to reduce the number of prohibitions between attribute levels. Parents were randomized to see 1 of 3 chances of winning the lottery: 1:10, 1:5, or 1:3 (eAppendix in the Supplement).

### Survey Measures

We obtained data on parent demographic characteristics (eg, race/ethnicity and sex) from self-reported data collected when parents enrolled in the AmeriSpeak panel. Data on each child’s age, sex, and race/ethnicity were assessed in the survey via parental report.

Data on parent health characteristics, including height, weight, and long-term health conditions, were obtained from self-reported data collected when parents enrolled in the AmeriSpeak panel. Data on the child’s height and weight were collected as previously described. Parents were also asked how they would characterize their child’s weight (ie, underweight, about the right weight, overweight).

The 8-item restriction factor of the Child Feeding Questionnaire was used to assess family conflict around food. This factor assesses how much parents have to regulate their child’s food consumption on a Likert scale.^31^ The survey also ascertained the child’s primary caregiver by asking how often the respondent was responsible for feeding the child at home, also on a Likert scale. Finally, parents were asked whether they had ever sought professional treatment for their child’s or their own weight.

### Statistical Analysis

Parents and children were classified as having healthy weight, being affected by overweight, or being affected by obesity in accordance with Centers for Disease Control and Prevention guidelines for adults and children.^32,33^ We calculated the relative importance of each attribute as the proportion of the sum of its utility score range, providing us with an understanding of the difference each attribute could make in the total utility of the program design. We used a hierarchical Bayesian logit model to estimate utilities for each attribute level. This model can estimate individual-level utilities, supporting analyses of preference heterogeneity and allowing for the use of survey weights to obtain population-level estimates.^34,35^ Utility values (UVs) are presented as 0 centered, in which all the values sum to 0 and are scaled so that ranges are comparable across respondents.^36^ Positive utilities that are significantly greater than 0 indicate that respondents were more likely to choose a profile with that level compared with the overall mean, and negative levels that are significantly lower than 0 indicate that respondents were less likely to choose a profile with that level compared with the overall mean. Larger utilities represent stronger preferences.

Utilities were used to calculate respondents’ willingness to pay for each nonmonetary attribute, ie, how much money each respondent would be willing to give up to receive an incentive program with their preferred attribute levels. Linear regressions were used to examine associations of utilities (dependent variable) with demographic and health characteristics (independent variables). Statistical significance was set at α = .05 following a 2-tailed test.

Analyses were conducted in Stata version 14 (StataCorp) and Sawtooth Software version 9.6.1 (Sawtooth Software Inc). Although the AmeriSpeak panel is a probability-based nationally representative sample, all analyses are weighted to more accurately represent the US population.

## Results

### Sample Characteristics

Based on a 33.7% initial recruitment rate, a profile completion rate of 88.1%, and a 15.8% invitation acceptance rate, the weighted cumulative response rate, as defined by AAPOR,^37^ for this study was 4.2%. Screening was completed by 819 parents of children aged 6 to 17 years in the AmeriSpeak panel in March 2018. Of those, 339 (41.4%) met child age and BMI percentile eligibility criteria, and 304 (89.7%) completed surveys. On average, the survey took 8.5 minutes to complete.

The sample was racially and ethnically diverse, with 53.3% of parents and 50.8% of children being non-Hispanic white. Among other sample demographic characteristics, 42.6% of parents had a household income less than $50 000, 28.3% of parents had completed a bachelor’s degree, and 74.5% of respondents were the primary caregiver for their child (Table 1).

**Table 1.** Population Demographic Characteristics of 304 Respondents

Characteristic	%^a^
Female	
Parent	59.4
Child	41.6
Parent age, y	
18-29	7.8
30-44	57.5
≥45	34.7
Child age, y	
6-12	57.2
13-17	42.8
Parent race/ethnicity	
White, non-Hispanic	53.3
Black, non-Hispanic	11.5
Hispanic	27.3
Other^b^	7.9
Child race/ethnicity	
White, non-Hispanic	50.8
Black, non-Hispanic	12.6
Hispanic	25.2
Other^b^	11.4
Region	
Northeast	16.9
Midwest	20.8
South	44.0
West	18.2
Income, $	
<50 000	42.6
50 000-99 999	32.8
≥100 000	24.6
Highest level of parent education	
<High school	11.3
High school	30.6
Some college	29.8
≥Bachelor’s degree	28.3
Married	70.0
Respondent is child’s primary caregiver^c^	74.5
Lives in metropolitan area	90.9
Household size, mean (SE)	4.4 (0.09)

^a^ All percentages represent weighted estimates.

^b^ Other category includes respondents who indicated that they were of 2 or more races, Asian, Native Hawaiian/Pacific Islander, or American Indian/Alaska Native.

^c^ Respondent was defined as the child’s primary caregiver if the respondent reported being responsible for feeding the child “most of the time” or “always.”

Most of the respondent parents (85.3%) had overweight or obesity. While all children had obesity based on parent report of child weight and height, only 65.3% of parents perceived their child to have obesity. Overall, 55.8% and 41.9% of parents and children, respectively, previously received treatment for their weight (Table 2).

**Table 2.** Family Health Characteristics and Behaviors of 304 Respondents

Characteristic	%^a^
Parent BMI class^b^	
Healthy weight	14.7
Overweight	26.4
Obese	58.9
Parent has any long-term health condition^c^	57.0
Child with obesity^b^	100
Child Feeding Questionnaire score, mean (SE)^d^	3.4 (0.77)
Parent perceives child as overweight or obese	65.3
Parent previously attempted weight loss	55.8
Child previously attempted weight loss	41.9

Abbreviation: BMI, body mass index (calculated as weight in kilograms divided by height in meters squared).

^a^ All percentages represent weighted estimates.

^b^ For adults, healthy weight represents a BMI less than 25, overweight represents a BMI between 25 and 30, and obese represents a BMI greater than 30. For children, healthy weight represents a BMI below the 85th percentile for age and sex, overweight represents a BMI between the 85th and 95th percentiles, and obese represents a BMI in or above the 95th percentile.

^c^ Parent reported a history of high cholesterol levels, high blood pressure, heart attack, coronary heart disease, stroke, diabetes, cancer, emphysema, asthma, chronic obstructive pulmonary disease, depression, and/or anxiety.

^d^ Mean score for 8 questions in the restriction factor of the Child Feeding Questionnaire. Responses to each question were provided on a Likert Scale and were scored as follows: 1, disagree; 2, slightly disagree; 3, neutral; 4, slightly agree; and 5, agree.

**Figure 2.**
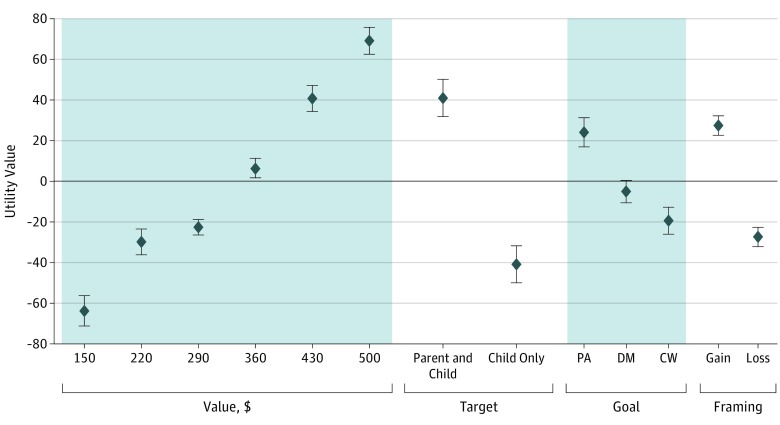
Discrete Choice Experiment Results CW indicates change in weight; DM, dietary monitoring; PA, physical activity; and error bars, 95% confidence intervals.

Parents had the strongest preferences for the monetary value of the incentive, with higher payments being preferred to lower payments. Utility values ranged from −63.8 (95% CI, −71.2 to −56.4) for a $150 incentive to 69.2 (95% CI, 62.5 to 75.9) for a $500 incentive. Next in importance was the incentive target. Parents significantly preferred financial incentives that targeted the parent and the child (UV, 40.9; 95% CI, 31.8 to 50.1) as opposed to the child only (UV, −40.9; 95% CI, −50.1 to −31.8). The next most important attribute was the payment structure. Parents preferred gain-framed payments (UV, 27.4; 95% CI, 22.6 to 32.2) to loss-framed payments (UV, −27.4; 95% CI, −32.2 to −22.6). Last, the goal being rewarded was the least important attribute, with physical activity being preferred (UV, 24.0; 95% CI, 16.8 to 31.2) compared with dietary monitoring (UV, −5.1; 95% CI, −10.6 to 0.4) or change in weight (UV, −18.9; 95% CI, −25.7 to −12.1) (Figure 2).

We calculated parents’ willingness to accept less money for more preferred attribute levels. Parents would be willing to accept lower-value incentives if both the parent and the child (vs the child alone) were incentivized to meet the goal (mean difference [MD], −$108; 95% CI, −$132 to −$84), if the financial incentive used a gain-framed payment structure (MD, −$72; 95% CI, −$85 to −$59), and if physical activity goals rather than weight loss (MD, −$63; 95% CI, −$82 to −$44) or dietary monitoring goals (MD, −$5; 95% CI, −$1 to $28) were incentivized. Overall, 20.6% of parents preferred a lottery system with a larger reward to a smaller, guaranteed reward. Preferences did not vary whether the chance of winning the lottery was 1:3, 1:5, or 1:10; 24.2% selected a lottery with a 1 in 10 chance of winning, 29.2% selected the 1:5 lottery, and 25.9% selected the 1:3 lottery (*P* = .26).

Some demographic and health characteristics, including parent and child race/ethnicity, parent and child sex, family income, and parent BMI, were associated with significant differences in utilities in univariate linear regressions. However, the overall ranking of attribute preferences remained constant for all subgroups.

## Discussion

In this study among a nationally representative sample of parents of children with obesity, a DCE assessed preferences for financial incentives that would motivate engagement with FBT for childhood obesity. Parents would be willing to accept lower-value incentives for an incentive program that targeted both the parent and child vs only the child, rewarded physical activity vs dietary monitoring or a change in weight, and used a gain-framed vs a loss-framed payment structure.

If incentives are unacceptable to their target populations, uptake of voluntary programs will remain low.^25^ Additionally, using preference-aligned incentives that parents think they can achieve may improve parent and child perceived self-efficacy. In turn, self-efficacy can influence the initiation and sustainability of behaviors in the face of adversity, thus improving FBT effectiveness and reducing program attrition.^38^

Dozens of financial incentive programs could be explored in randomized clinical trials (RCTs), varying the type of goal being rewarded, the structure of the payment, the value of the reward, the frequency of payment, the type of reward (cash vs noncash), and the person who has to meet the goal.^39^ Rather than evaluating the association of every combination of incentive attribute levels, a DCE can be used to narrow the number of options that warrant evaluation in RCTs. To our knowledge, only 3 studies have used DCEs to assess preferences for obesity-related incentives, all for adults.^24,25,26^ Two UK-based studies (by Promberger et al^24^ and Giles et al^25^) assessed the acceptability of using incentives to encourage healthy behaviors in adults. The focus on acceptability is natural for a nationalized health care system in which citizens bear the cost of incentive programs. Both studies found weight loss incentives to be publicly acceptable. Another study by Farooqui et al^26^ used a DCE to assess preferences for and likely uptake of a physical activity intervention targeting older adults in Singapore. They found that a $500 incentive over 6 months would improve program uptake.

The US health care system supports health behavior incentive programs among publicly^40^ and privately^41^ insured populations, so this study focused on preferences among a population who would be eligible to receive incentives rather than on public opinion about incentives. However, we did not explicitly assess likely program uptake like Farooqui et al,^26^ which is an area for future research. Still, the present study is one of the first to assess preferences for childhood obesity−related incentive programs, including preferences for payment structures and incentives that monitor obesity-related outcomes other than physical activity.

We calculated parents’ willingness to trade higher-value incentives for preferred attribute levels based on incentive values ranging from $150 to $500 over 6 months. This range of incentive program values was informed by qualitative interviews^6^ and the annual health care costs attributable to childhood obesity^42^; it is similar to the value of incentives evaluated by Farooqui et al.^26^ However, health care payers may be willing to provide incentives above $500, given that parents and children can see improvements in health as well as health care use and that health care expenditures attributable to obesity are higher for adults than children.^43^ An economic evaluation can assess payers’ return on investment and the ideal maximum incentive program value.

Interestingly, there is a mismatch between parent-stated preferences for FBT incentive programs and presumed best practices from behavioral economic and behavior change perspectives. Behavioral economic theory and empirical evidence support the use of loss-framed incentives and lotteries for motivating behavior change. These incentive designs use loss aversion, where individuals strive to prevent losses rather than obtain equivalent gains.^44^ Incentive designs also capitalize on people’s tendencies to overestimate their probability of winning lotteries.^45^ Recent studies with adult and pediatric populations suggest loss-framed and lottery-based payment schemes are effective for improving health behaviors,^14,15,19,46^ although, regardless of their framing, financial incentive programs are not always sustainable. After incentives end, there is sometimes no difference between intervention and control groups in adherence to healthy behaviors.^14,15,46^

An RCT should assess whether incentives that are aligned with patient preferences create more sustainable behavior change than those that are not and should also assess the mediating pathways (eg, self-efficacy). Moreover, an RCT that uses a crossover study can reveal how changing the incentive structure over time might affect adherence.

From a pediatric perspective, the use of loss-framed financial incentives and lottery-based incentives may not be ideal. Children are still developing competence in self-care skills^27^ and may be demoralized by loss-framed rewards when they do not to meet goals, especially since goal attainment may be out of the child’s control. Children may also be demoralized when they meet their goals but do not win a lottery. Additionally, any extrinsic incentives, no matter the design, can potentially negatively impact a child’s intrinsic motivation, which parents are eager to protect.^6,47^

From a behavior change perspective, it is more appropriate and effective to incentivize behaviors over which parents and children have control, such as physical activity or dietary monitoring, rather than to incentivize outcomes, such as a change in weight. The ability to engage in dietary monitoring is associated with treatment success^48,49,50^ and is more strongly associated with success in FBT than physical activity. Our study results, which showed that parents prefer programs that reward physical activity over dietary monitoring, may stem from recent trends in the use of wearable fitness trackers.^51^ Parents may have higher perceived efficacy for meeting physical activity vs dietary monitoring goals. Finally, parents may mistakenly believe that physical activity will lead to weight loss, ignoring the effect of compensatory behaviors, such as increased dietary consumption after exercising.^52,53^ Future research should assess perceived barriers to parents’ engaging in dietary monitoring.

Although we collected data on a wide range of characteristics in a diverse sample, we found no significant associations of preferences with parent demographic and health characteristics. Discrete choice experiments could be used to identify heterogeneity in preferences and could therefore be used to develop personalized incentive programs. Conducting this survey with a larger sample may facilitate such analyses and is another area for future research.

### Limitations

This study was subject to limitations. We were unable to include more incentive attributes because of the limitations inherent in any DCE, which restricts the total number of attributes and levels that can be reasonably included while still maintaining respondent comprehension and data quality.^30^ A partial profile design could be used but would require a substantial increase in sample size to produce valid estimates. This DCE focused on the most critical attributes of FBT incentives.

Our questions regarding the lottery only examined a 1-time drawing and were not as nuanced as lotteries that have been evaluated in the experimental literature.^14,15^ For example, parents may have different preferences for daily or weekly lotteries.

Conducting this survey online allowed us to reach a nationally representative, generalizable sample but therefore relied on parent-reported height and weight. Owing to misreporting of child height and weight, some parents may have been inappropriately considered ineligible to complete the survey.^54^

This study addressed only incentives that have a monetary value. Other incentives, including social incentives, could also influence program adherence and were not explicitly explored. Also, the survey was only fielded in English, and findings may not generalize to other language groups.

## Conclusions

In this survey study of 304 parents of children with obesity, participants chose incentives that offered higher payments but were willing to accept lower amounts if the incentives used positive reinforcement, targeted both parents and children, and rewarded physical activity. Incentives could improve enrollment in and reduce attrition from FBT programs for childhood obesity. Parent preferences for the design of incentives differ from behavioral economic and behavior change theory. It is unclear whether the incentive that would reduce attrition and maximize the effectiveness of FBT should align with researcher best practices or with parent preferences. An RCT that evaluates the efficacy and cost-effectiveness of different FBT incentive designs is needed.

## Notes

Supplement. **eAppendix.** Survey to Assess Parents’ Preferences for Incentives in Family-Based Childhood Obesity Treatment Programs

## References

[1] Grossman DC, Bibbins-Domingo K, Curry SJ, ; US Preventive Services Task Force Screening for obesity in children and adolescents: US Preventive Services Task Force recommendation statement. JAMA. 2017;317(23):-. doi:10.1001/jama.2017.6803 28632874

[2] Wilfley DE, Tibbs TL, Van Buren DJ, Reach KP, Walker MS, Epstein LH Lifestyle interventions in the treatment of childhood overweight: a meta-analytic review of randomized controlled trials. Health Psychol. 2007;26(5):521-532. doi:10.1037/0278-6133.26.5.521 17845100 PMC2040042

[3] Kelleher E, Davoren MP, Harrington JM, Shiely F, Perry IJ, McHugh SM Barriers and facilitators to initial and continued attendance at community-based lifestyle programmes among families of overweight and obese children: a systematic review. Obes Rev. 2017;18(2):183-194. doi:10.1111/obr.12478 27862851 PMC5245104

[4] Skelton JA, Beech BM Attrition in paediatric weight management: a review of the literature and new directions. Obes Rev. 2011;12(5):e273-e281. doi:10.1111/j.1467-789X.2010.00803.x 20880126 PMC3079805

[5] Skelton JA, Martin S, Irby MB Satisfaction and attrition in paediatric weight management. Clin Obes. 2016;6(2):143-153. doi:10.1111/cob.12138 27008068 PMC9093167

[6] Jacob-Files E, Powell J, Wright DR Exploring parent attitudes around using incentives to promote engagement in family-based weight management programs. Prev Med Rep. 2018;10:278-284. doi:10.1016/j.pmedr.2018.04.007 29868380 PMC5984230

[7] Staiano AE, Marker AM, Comeaux J, Frelier JM, Hsia DS, Broyles ST Family-based behavioral treatment for childhood obesity: caretaker-reported barriers and facilitators. Ochsner J. 2017;17(1):83-92. 28331454 PMC5349643

[8] Grow HM, Hsu C, Liu LL, Understanding family motivations and barriers to participation in community-based programs for overweight youth: one program model does not fit all. J Public Health Manag Pract. 2013;19(4):e1-e10. doi:10.1097/PHH.0b013e31825ceaf9 23328502

[9] Wilfley DE, Staiano AE, Altman M, ; Improving Access and Systems of Care for Evidence-Based Childhood Obesity Treatment Conference Workgroup Improving access and systems of care for evidence-based childhood obesity treatment: conference key findings and next steps. Obesity (Silver Spring). 2017;25(1):16-29. doi:10.1002/oby.21712 27925451 PMC5373656

[10] Janicke DM, Steele RG, Gayes LA, Systematic review and meta-analysis of comprehensive behavioral family lifestyle interventions addressing pediatric obesity. J Pediatr Psychol. 2014;39(8):809-825. doi:10.1093/jpepsy/jsu023 24824614

[11] Theim KR, Sinton MM, Goldschmidt AB, Adherence to behavioral targets and treatment attendance during a pediatric weight control trial. Obesity (Silver Spring). 2013;21(2):394-397. doi:10.1002/oby.20281 23532993 PMC3410964

[12] Mantzari E, Vogt F, Shemilt I, Wei Y, Higgins JP, Marteau TM Personal financial incentives for changing habitual health-related behaviors: a systematic review and meta-analysis. Prev Med. 2015;75:75-85. doi:10.1016/j.ypmed.2015.03.001 25843244 PMC4728181

[13] Finkelstein EA, Brown DS, Brown DR, Buchner DM A randomized study of financial incentives to increase physical activity among sedentary older adults. Prev Med. 2008;47(2):182-187. doi:10.1016/j.ypmed.2008.05.002 18571226

[14] Patel MS, Asch DA, Rosin R, Framing financial incentives to increase physical activity among overweight and obese adults: a randomized, controlled trial. Ann Intern Med. 2016;164(6):385-394. doi:10.7326/M15-1635 26881417 PMC6029433

[15] Patel MS, Volpp KG, Rosin R, A randomized, controlled trial of lottery-based financial incentives to increase physical activity among overweight and obese adults. Am J Health Promot. 2018;32(7):1568-1575. doi:10.1177/0890117118758932 29534597

[16] Kane RL, Johnson PE, Town RJ, Butler M A structured review of the effect of economic incentives on consumers’ preventive behavior. Am J Prev Med. 2004;27(4):327-352. doi:10.1016/j.amepre.2004.07.002 15488364

[17] Mitchell MS, Goodman JM, Alter DA, Financial incentives for exercise adherence in adults: systematic review and meta-analysis. Am J Prev Med. 2013;45(5):658-667. doi:10.1016/j.amepre.2013.06.017 24139781

[18] Shin DW, Yun JM, Shin JH, Enhancing physical activity and reducing obesity through Smartcare and financial incentives: a pilot randomized trial. Obesity (Silver Spring). 2017;25(2):302-310. doi:10.1002/oby.21731 28063226

[19] Volpp KG, John LK, Troxel AB, Norton L, Fassbender J, Loewenstein G Financial incentive-based approaches for weight loss: a randomized trial. JAMA. 2008;300(22):2631-2637. doi:10.1001/jama.2008.804 19066383 PMC3583583

[20] Jeffery RW Financial incentives and weight control. Prev Med. 2012;55(suppl):S61-S67. doi:10.1016/j.ypmed.2011.12.024 22244800 PMC3342479

[21] O’Donoghue T, Rabin M The economics of immediate gratification. J Behav Decis Making. 2000;13(2):233-250. doi:10.1002/(SICI)1099-0771(200004/06)13:2<233::AID-BDM325>3.0.CO;2-U

[22] Kullgren JT, Troxel AB, Loewenstein G, Individual- versus group-based financial incentives for weight loss: a randomized, controlled trial. Ann Intern Med. 2013;158(7):505-514. doi:10.7326/0003-4819-158-7-201304020-00002 23546562 PMC3994977

[23] VanEpps EM, Troxel AB, Villamil E, Financial incentives for chronic disease management: results and limitations of 2 randomized clinical trials with New York Medicaid patients. Am J Health Promot. 2018;32(7):1537-1543. doi:10.1177/0890117117753986 29390862 PMC7256969

[24] Promberger M, Dolan P, Marteau TM “Pay them if it works”: discrete choice experiments on the acceptability of financial incentives to change health related behaviour. Soc Sci Med. 2012;75(12):2509-2514. doi:10.1016/j.socscimed.2012.09.033 23102753 PMC3686527

[25] Giles EL, Becker F, Ternent L, Sniehotta FF, McColl E, Adams J Acceptability of financial incentives for health behaviours: a discrete choice experiment. PLoS One. 2016;11(6):e0157403. doi:10.1371/journal.pone.0157403 27314953 PMC4912063

[26] Farooqui MA, Tan YT, Bilger M, Finkelstein EA Effects of financial incentives on motivating physical activity among older adults: results from a discrete choice experiment. BMC Public Health. 2014;14:141. doi:10.1186/1471-2458-14-141 24512102 PMC3933254

[27] Davis MM, Gance-Cleveland B, Hassink S, Johnson R, Paradis G, Resnicow K Recommendations for prevention of childhood obesity. Pediatrics. 2007;120(suppl 4):S229-S253. doi:10.1542/peds.2007-2329E 18055653

[28] Shartzer A, Johnston E The Survey of Family Planning and Women’s Lives: Methodology. Washington, DC: The Urban Institute; 2016.

[29] Wright DR Assessment of Parents’ Preferences for Incentives to Promote Engagement in Family Based Childhood Obesity Treatment. Cambridge, MA: Harvard Dataverse; 2018. https://dataverse.harvard.edu/dataset.xhtml?persistentId=doi:10.7910/DVN/NR4USF. Accessed February 25, 2019. 10.1001/jamanetworkopen.2019.1490 PMC6450425 30924902

[30] Bridges JF, Hauber AB, Marshall D, Conjoint analysis applications in health—a checklist: a report of the ISPOR Good Research Practices for Conjoint Analysis Task Force. Value Health. 2011;14(4):403-413. doi:10.1016/j.jval.2010.11.013 21669364

[31] Birch LL, Fisher JO, Grimm-Thomas K, Markey CN, Sawyer R, Johnson SL Confirmatory factor analysis of the Child Feeding Questionnaire: a measure of parental attitudes, beliefs and practices about child feeding and obesity proneness. Appetite. 2001;36(3):201-210. doi:10.1006/appe.2001.0398 11358344

[32] Centers for Disease Control and Prevention About adult BMI. https://www.cdc.gov/healthyweight/assessing/bmi/adult_bmi/. Accessed February 21, 2019.

[33] Centers for Disease Control and Prevention About child and teen BMI. https://www.cdc.gov/healthyweight/assessing/bmi/childrens_BMI/about_childrens_BMI.html. Accessed February 21, 2019.

[34] Hauber AB, González JM, Groothuis-Oudshoorn CG, Statistical methods for the analysis of discrete choice experiments: a report of the ISPOR Conjoint Analysis Good Research Practices Task Force. Value Health. 2016;19(4):300-315. doi:10.1016/j.jval.2016.04.004 27325321

[35] Lichtenstein GR, Waters HC, Kelly J, Assessing drug treatment preferences of patients with Crohn’s disease: a conjoint analysis. Patient. 2010;3(2):113-123. doi:10.2165/11314880-000000000-00000

[36] Orme B Getting Started With Conjoint Analysis: Strategies for Product Design and Pricing Research. 2nd ed Madison, WI: Research Publishers LLC; 2010.

[37] American Association for Public Opinion Research Standard definitions: final dispositions of case codes and outcome rates for surveys. https://www.aapor.org/AAPOR_Main/media/publications/Standard-Definitions20169theditionfinal.pdf. Accessed February 25, 2019.

[38] Bandura A Self-efficacy: toward a unifying theory of behavioral change. Psychol Rev. 1977;84(2):191-215. doi:10.1037/0033-295X.84.2.191 847061

[39] Adams J, Giles EL, McColl E, Sniehotta FF Carrots, sticks and health behaviours: a framework for documenting the complexity of financial incentive interventions to change health behaviours. Health Psychol Rev. 2014;8(3):286-295. doi:10.1080/17437199.2013.848410 25053215

[40] Medicare program: final waivers in connection with the Shared Savings Program: final rule. Fed Regist. 2015;80(209):66725-66745. 26524770

[41] Madison K, Schmidt H, Volpp KG Smoking, obesity, health insurance, and health incentives in the Affordable Care Act. JAMA. 2013;310(2):143-144. doi:10.1001/jama.2013.7617 23765171

[42] Wright DR, Prosser LA The impact of overweight and obesity on pediatric medical expenditures. Appl Health Econ Health Policy. 2014;12(2):139-150. doi:10.1007/s40258-014-0088-7 24652198

[43] Finkelstein EA, Trogdon JG, Cohen JW, Dietz W Annual medical spending attributable to obesity: payer- and service-specific estimates. Health Aff (Millwood). 2009;28(5):w822-w831. doi:10.1377/hlthaff.28.5.w822 19635784

[44] Kahneman D, Tversky A Prospect theory: an analysis of decision under risk. Econometrica. 1979;47(2):263-291. doi:10.2307/1914185

[45] Ungemach C, Chater N, Stewart N Are probabilities overweighted or underweighted when rare outcomes are experienced (rarely)? Psychol Sci. 2009;20(4):473-479. doi:10.1111/j.1467-9280.2009.02319.x 19399978

[46] Wong CA, Miller VA, Murphy K, Effect of financial incentives on glucose monitoring adherence and glycemic control among adolescents and young adults with type 1 diabetes: a randomized clinical trial. JAMA Pediatr. 2017;171(12):1176-1183. doi:10.1001/jamapediatrics.2017.3233 29059263 PMC6583649

[47] Deci EL, Koestner R, Ryan RM A meta-analytic review of experiments examining the effects of extrinsic rewards on intrinsic motivation. Psychol Bull. 1999;125(6):627-668. doi:10.1037/0033-2909.125.6.627 10589297

[48] Tsai AG, Fabricatore AN, Wadden TA, Readiness redefined: a behavioral task during screening predicted 1-year weight loss in the Look AHEAD study. Obesity (Silver Spring). 2014;22(4):1016-1023. doi:10.1002/oby.20648 24151217 PMC4109684

[49] Saelens BE, McGrath AM Self-monitoring adherence and adolescent weight control efficacy. Child Health Care. 2003;32(2):137-152. doi:10.1207/S15326888CHC3202_5

[50] Mockus DS, Macera CA, Wingard DL, Peddecord M, Thomas RG, Wilfley DE Dietary self-monitoring and its impact on weight loss in overweight children. Int J Pediatr Obes. 2011;6(3-4):197-205. doi:10.3109/17477166.2011.590196 21722068 PMC3788598

[51] Gonzalez R Science says fitness trackers don’t work: wear one anyway. *Wired* December 25, 2017. https://www.wired.com/story/science-says-fitness-trackers-dont-work-wear-one-anyway/. Accessed February 21, 2019.

[52] Melanson EL, Keadle SK, Donnelly JE, Braun B, King NA Resistance to exercise-induced weight loss: compensatory behavioral adaptations. Med Sci Sports Exerc. 2013;45(8):1600-1609. doi:10.1249/MSS.0b013e31828ba942 23470300 PMC3696411

[53] Mura G, Rocha NB, Helmich I, Physical activity interventions in schools for improving lifestyle in European countries. Clin Pract Epidemiol Ment Health. 2015;11(suppl 1 M5):77-101. 25834629 10.2174/1745017901511010077 PMC4378026

[54] O’Connor DP, Gugenheim JJ Comparison of measured and parents’ reported height and weight in children and adolescents. Obesity (Silver Spring). 2011;19(5):1040-1046. doi:10.1038/oby.2010.278 21127476

