# Supplementary material for: Assessment of Parents’ Preferences for Incentives to Promote Engagement in Family-Based Childhood Obesity Treatment
Source: JAMA Netw Open. 2019 Mar 29;2(3):e191490. doi: 10.1001/jamanetworkopen.2019.1490 (PMC6450425; doi:10.1001/jamanetworkopen.2019.1490)
Supplement: Supplement. — eAppendix. Survey to Assess Parents’ Preferences for Incentives in Family-Based Childhood Obesity Treatment Programs [file jamanetwopen-2-e191490-s001.pdf]

Supplementary Online Content

Wright DR, Saelens BE, Fontes A, Lavelle TA. Assessment of parents' preferences for incentives to promote engagement in family-based childhood obesity treatment. *JAMA Netw Open.* 2019;2(3):e191490. doi:10.1001/jamanetworkopen.2019.1490

eAppendix. Survey to Assess Parents' Preferences for Incentives in Family-Based Childhood Obesity Treatment Programs

This supplementary material has been provided by the authors to give readers additional information about their work.

© 2019 Wright DR et al. *JAMA Network Open.*

eAppendix: Survey to Assess Parents' Preferences for Incentives in Family-Based Childhood Obesity Treatment Programs

INFORMATION SHEET

You have the option to take part in a research study called Leveraging Incentives for Obesity Prevention (LION). The goals of this form are to give you information about what would happen if you choose to take part in the study and to help you decide if you want to be in the study.

Please review the following information about this study:

1) The goal of this research study is to figure out what might motivate families to participate in a family-based childhood weight management program.

2) If you agree to participate in this study, you will be asked to complete a 15 minute-long survey administered by AmeriSpeak. We will ask you some questions about the age, gender, height, and weight for one of your children who may have a high weight status. It may be helpful for you to look up recent measurement information if you can.

3) We will obtain data from your AmeriSpeak profile including information on your age, gender, level of education, race/ethnicity, household income, household size, employment status, census region, and health status.

4) We will not be able to identify you or your child(ren) from the survey or profile data, and so there are minimal concerns about a potential loss of privacy and/or confidentiality.

5) We do not expect this study to directly benefit you or your child(ren). We hope to use information from this survey to benefit families of children with a high weight status.

6) This survey is completely voluntary. You can choose to stop taking the survey at any time.

7) If, at any time, you have questions about your rights as a research participant, or if you have questions, concerns or complaints about the survey, you may contact:

Davene Wright, PhD
LION Principal Investigation
University of Washington
Seattle Children's Research Institute
davene.wright@seattlechildrens.org
phone: 206-884-8241

Seattle Children's Hospital
Institutional Review Board
Re: LION (STUDY #0717)
irb@seattlechildrens.org
phone: 206-987-7804

© 2019 Wright DR et al. *JAMA Network Open.*

Q0.
Are you a parent or legal guardian of a child aged 6-17 years?

1. Yes
2. No

TERMINATE IF NO OR MISSING

As we mentioned earlier, the goal of this research study is to figure out what might motivate families to participate in a family-based childhood weight management program. We'll be asking some questions about you and one of your children aged 6-17 years who may be overweight. If you have more than one child who is 6-17 years old who fits this description, please think of the child with the most recent birthday.

Q1_AGE.
How old is your child?

_____ **[NUM BOX, RANGE 6-17]**

[IF Q1_AGE=REFUSED, TERMINATE]

Q1_SEX.
What sex was this child assigned at birth?

1. Male
2. Female

[IF Q1_SEX=REFUSED, TERMINATE]

Q2a.
How tall is your child without shoes?

_____ **[NUM BOX, RANGE 3-7]** feet _____ **[NUM BOX, RANGE 0-11]** inches

[PROMPT ONCE AND TERMINATE IF BOTH Q2A_FEET AND Q2A_INCHES REFUSED]

Q2b.
How much does your child weigh without shoes?

_____ **[NUM BOX, RANGE 30 TO 400]** pounds

[PROMPT ONCE AND TERMINATE IF REFUSED]

© 2019 Wright DR et al. *JAMA Network Open.*

IF INELIGIBLE DUE TO BMI < 95th PERCENTILE:
Thank you for your time today. Unfortunately you are not eligible for this study. We value your opinion and hope that you will participate in future AmeriSpeak surveys. Thank you for your time today.

We will redirect you to the AmeriSpeak Member Portal in [n] seconds.

Q3.
Imagine that you could participate in a family-based treatment program to help you manage your child's weight. As part of the program, to be successful, you will have to:

- attend weekly in-person meetings with your child,
- track the food you and your child eat at every meal,
- change the types of food you serve your family,
- give your child the opportunity to have 60 minutes of outdoor play or physical activity a day,
- and limit your child's access to screen time (ex. video games).

You will be reimbursed for any gas, toll, and parking costs. Parents who participate in a program like this spend approximately 5 hours per week for 6 months performing the previously described program activities. Many children and parents who participate in such programs see an improvement in weight and quality of life.

As a bonus, your health provider is considering giving you a reward to motivate program participation. You will be given points if you meet certain goals, like meeting a daily physical activity goal or keeping an honest daily record of what foods you and your child eat. Points can be exchanged for prizes, including cash, a gift card, or a non-monetary gift (ex. a tablet or sports equipment), online. You can keep the points for yourself or share them with your family as you see fit.

The rewards have different characteristics, all of which are listed below. We want to know which of the two rewards would motivate you more. There are no right or wrong answers.

Let's do a warm-up exercise. Take a close look at the characteristics of each reward described below. Which of these two rewards would motivate you more?

**Reward A**	**Characteristics**	**Reward B**
You start with nothing and earn points for each goal you meet, up to $100 worth of points	**Payment structure and total value of the reward over 6 months**	You start with nothing and earn points for each goal you meet, up to $300 worth of points
Weekly physical activity time, measured using a Fitbit or smartwatch	**Goal that is being rewarded**	Weekly physical activity time, measured using a Fitbit or smartwatch
Parent and child	**Who has to meet goal to earn reward**	Parent and child
☐ **I prefer reward A**		☐ **I prefer reward B**

© 2019 Wright DR et al. *JAMA Network Open.*

[SHOW IF Q3=1 (reward A)]
Q4.
Great! Here's another one. Imagine that you could participate in a family-based treatment program to help you manage your child's weight. As part of the program, to be successful, you will have to:

- attend weekly in-person meetings with your child,
- track the food you and your child eat at every meal,
- change the types of food you serve your family,
- give your child the opportunity to have 60 minutes of outdoor play or physical activity a day,
- and limit your child's access to screen time (ex. video games).

You will be reimbursed for any gas, toll, and parking costs. Parents who participate in a program like this spend approximately 5 hours per week for 6 months performing the previously described program activities. Many children and parents who participate in such programs see an improvement in weight and quality of life.

As a bonus, your health provider is considering giving you a reward to motivate program participation. You will be given points if you meet certain goals, like meeting a daily physical activity goal or keeping an honest daily record of what foods you and your child eat. Points can be exchanged for prizes, including cash, a gift card, or a non-monetary gift (ex. a tablet or sports equipment), online. You can keep the points for yourself or share them with your family as you see fit.

The rewards have different characteristics, all of which are listed below. We want to know which of the two rewards would motivate you more. There are no right or wrong answers.

Think about all of the characteristics of each reward and whether or how they are different. Which reward would you find more motivating?

**Reward A**	**Characteristics**	**Reward B**
You start with nothing and earn points for each goal you meet, up to $300 worth of points	**Payment structure and total value of the reward over 6 months**	You start with nothing and earn points for each goal you meet, up to $200 worth of points
Weekly physical activity time, measured using a Fitbit or smartwatch	**Goal that is being rewarded**	Weekly physical activity time, measured using a Fitbit or smartwatch
Parent and child	**Who has to meet goal to earn reward**	Parent and child
☐ **I prefer reward A**		☐ **I prefer reward B**

© 2019 Wright DR et al. *JAMA Network Open.*

DISCRETE CHOICE EXERCISE Q6_1 to Q6_10) USES DESIGN FILE:
LION_DiscreteChoiceDesign.xlsx

Q6_1.
Imagine that you could participate in a family-based treatment program to help you manage your child's weight. As part of the program, to be successful, you will have to:

- attend weekly in-person meetings with your child,
- track the food you and your child eat at every meal,
- change the types of food you serve your family,
- give your child the opportunity to have 60 minutes of outdoor play or physical activity a day,
- and limit your child's access to screen time (ex. video games).

You will be reimbursed for any gas, toll, and parking costs. Parents who participate in a program like this spend approximately 5 hours per week for 6 months performing the previously described program activities. Many children and parents who participate in such programs see an improvement in weight and quality of life.

As a bonus, your health provider is considering giving you a reward to motivate program participation. You will be given points if you meet certain goals, like meeting a daily physical activity goal or keeping an honest daily record of what foods you and your child eat. Points can be exchanged for prizes, including cash, a gift card, or a non-monetary gift (ex. a tablet or sports equipment), online. You can keep the points for yourself or share them with your family as you see fit.

The rewards have different characteristics, all of which are listed below. We want to know which of the two rewards would motivate you more. There are no right or wrong answers.
[SPACE]
Looks like you have the hang of it! Keep going. Let us know which of the two possible rewards would motivate you in a family-based weight management program. There are no right or wrong answers.

[insert TASK 1 values below according to discrete choice design file]

Reward A	Characteristics	Reward B
[Concept 1, attribute A/B]	**Payment structure and total value of the reward over 6 months**	[Concept 2, attribute A/B]
[Concept 1, attribute C]	**Goal that is being rewarded**	[Concept 2, attribute C]
[Concept 1, attribute D]	**Who has to meet goal to earn reward**	[Concept 2, attribute D]
☐ **I prefer reward A**		☐ **I prefer reward B**

1 of 10

© 2019 Wright DR et al. *JAMA Network Open.*

Q6_2.
Great! Again, let us know which of the two possible rewards would motivate you in a family-based weight management program. There are no right or wrong answers.

[insert TASK 2 values below according to discrete choice design file]

Reward A	Characteristics	Reward B
[Concept 1, attribute A/B]	**Payment structure and total value of the reward over 6 months**	[Concept 2, attribute A/B]
[Concept 1, attribute C]	**Goal that is being rewarded**	[Concept 2, attribute C]
[Concept 1, attribute D]	**Who has to meet goal to earn reward**	[Concept 2, attribute D]
☐ **I prefer reward A**		☐ **I prefer reward B**

2 of 10

Q6_3.
What if the reward were structured a bit differently? Would one reward motivate you more than another? There are no right or wrong answers.

[insert TASK 3 values below according to discrete choice design file]

Reward A	Characteristics	Reward B
[Concept 1, attribute A/B]	**Payment structure and total value of the reward over 6 months**	[Concept 2, attribute A/B]
[Concept 1, attribute C]	**Goal that is being rewarded**	[Concept 2, attribute C]
[Concept 1, attribute D]	**Who has to meet goal to earn reward**	[Concept 2, attribute D]
☐ **I prefer reward A**		☐ **I prefer reward B**

3 of 10

© 2019 Wright DR et al. *JAMA Network Open.*

Q6_4.
Super! The reward characteristics are slightly different now. Which of the two rewards would motivate you more? There are no right or wrong answers.

[insert TASK 4 values below according to discrete choice design file]

Reward A	Characteristics	Reward B
[Concept 1, attribute A/B]	**Payment structure and total value of the reward over 6 months**	[Concept 2, attribute A/B]
[Concept 1, attribute C]	**Goal that is being rewarded**	[Concept 2, attribute C]
[Concept 1, attribute D]	**Who has to meet goal to earn reward**	[Concept 2, attribute D]
☐ **I prefer reward A**		☐ **I prefer reward B**

4 of 10

Q6_5.
How about now? Remember, there are no right or wrong answers, we just want to know your preference.

[insert TASK 5 values below according to discrete choice design file]

Reward A	Characteristics	Reward B
[Concept 1, attribute A/B]	**Payment structure and total value of the reward over 6 months**	[Concept 2, attribute A/B]
[Concept 1, attribute C]	**Goal that is being rewarded**	[Concept 2, attribute C]
[Concept 1, attribute D]	**Who has to meet goal to earn reward**	[Concept 2, attribute D]
☐ **I prefer reward A**		☐ **I prefer reward B**

5 of 10

© 2019 Wright DR et al. *JAMA Network Open.*

Q6_6.
You're more than halfway there! Which of the two rewards listed below would motivate you more. There are no right or wrong answers.

[insert TASK 1 values below according to discrete choice design file]

Reward A	Characteristics	Reward B
[Concept 1, attribute A/B]	**Payment structure and total value of the reward over 6 months**	[Concept 2, attribute A/B]
[Concept 1, attribute C]	**Goal that is being rewarded**	[Concept 2, attribute C]
[Concept 1, attribute D]	**Who has to meet goal to earn reward**	[Concept 2, attribute D]
☐ **I prefer reward A**		☐ **I prefer reward B**

6 of 10

Q6_7.
Great! These rewards have a few new options. Is there one reward you would prefer over the other?

[insert TASK 1 values below according to discrete choice design file]

Reward A	Characteristics	Reward B
Concept 1, attribute A/B	**Payment structure and total value of the reward over 6 months**	Concept 2, attribute A/B
Concept 1, attribute C	**Goal that is being rewarded**	Concept 2, attribute C
Concept 1, attribute D	**Who has to meet goal to earn reward**	Concept 2, attribute D
☐ **I prefer reward A**		☐ **I prefer reward B**

7 of 10

Q6_8.
Keep going! The rewards have different characteristics, all of which are listed below. We want to know which of the two rewards would motivate you more. There are no right or wrong answers.

[insert TASK 1 values below according to discrete choice design file]

Reward A	Characteristics	Reward B
Concept 1, attribute A/B	**Payment structure and total value of the reward over 6 months**	Concept 2, attribute A/B
Concept 1, attribute C	**Goal that is being rewarded**	Concept 2, attribute C
Concept 1, attribute D	**Who has to meet goal to earn reward**	Concept 2, attribute D
☐ **I prefer reward A**		☐ **I prefer reward B**

8 of 10

© 2019 Wright DR et al. *JAMA Network Open.*

Q6_9.
Let us know which of the two possible rewards would motivate you in a family-based weight management program. There are no right or wrong answers.

[insert TASK 9 values below according to discrete choice design file]

Reward A	Characteristics	Reward B
Concept 1, attribute A/B	**Payment structure and total value of the reward over 6 months**	Concept 2, attribute A/B
Concept 1, attribute C	**Goal that is being rewarded**	Concept 2, attribute C
Concept 1, attribute D	**Who has to meet goal to earn reward**	Concept 2, attribute D
☐ **I prefer reward A**		☐ **I prefer reward B**

9 of 10

Q6_10.
This is the last choice you have to make! Which one of these rewards would motivate you more?

[insert TASK 10 values below according to discrete choice design file]

Reward A	Characteristics	Reward B
Concept 1, attribute A/B	**Payment structure and total value of the reward over 6 months**	Concept 2, attribute A/B
Concept 1, attribute C	**Goal that is being rewarded**	Concept 2, attribute C
Concept 1, attribute D	**Who has to meet goal to earn reward**	Concept 2, attribute D
☐ **I prefer reward A**		☐ **I prefer reward B**

10 of 10

© 2019 Wright DR et al. *JAMA Network Open.*

[SHOW IF RND_00=0]

Q7a.
Now, imagine that instead of the scenario where you earn points if you meet your goals, you were entered into a drawing where you have the chance to win a reward if you meet your goals. If given a choice, which of the following rewards would motivate you more?

**Reward A**	**Reward B**
A certainty of earning up to $300 worth of points if you meet the goals	A lottery, with a minimum 1 in 10 chance of earning points worth $3,000 if you meet the goal
☐ **I prefer reward A**	☐ **I prefer reward B**

[SHOW IF RND_00=1]

Q7B.
Now, imagine that instead of the scenario where you earn points if you meet your goals, you were entered into a drawing where you have the chance to win a reward if you meet your goals. If given a choice, which of the following rewards would motivate you more?

**Reward A**	**Reward B**
A certainty of earning up to $300 worth of points if you meet the goals	A lottery, with a minimum 1 in 5 chance of earning points worth $1,500 if you meet the goal
☐ **I prefer reward A**	☐ **I prefer reward B**

[SHOW IF RND_00=2]

Q7C.
Now, imagine that instead of the scenario where you earn points if you meet your goals, you were entered into a drawing where you have the chance to win a reward if you meet your goals. If given a choice, which of the following rewards would motivate you more?

**Reward A**	**Reward B**
A certainty of earning up to $300 worth of points if you meet the goals	A lottery, with a minimum 1 in 3 chance of earning points worth $900 if you meet the goal
☐ **I prefer reward A**	☐ **I prefer reward B**

© 2019 Wright DR et al. *JAMA Network Open.*

Q8.
How much do you agree or disagree with the following statements:

		1<br>Disagree	2<br>Slightly disagree	3<br>Neutral	4<br>Slightly agree	5<br>Agree
A	I have to be sure that my child does not eat to many sweets (candy, ice cream, cake, pastries)	☐	☐	☐	☐	☐
B	I have to be sure that my child does not eat too many high-fat foods	☐	☐	☐	☐	☐
C	I have to be sure that my child does not eat too much of his/her favorite foods	☐	☐	☐	☐	☐
D	I intentionally keep some foods out of my child's reach	☐	☐	☐	☐	☐
E	I offer sweets (candy, ice cream, cake, pastries) to my child as a reward for good behavior	☐	☐	☐	☐	☐
F	I offer my child his/her favorite foods in exchange for good behavior	☐	☐	☐	☐	☐
G	If I did not guide or regulate my child's eating he/she would eat too many junk foods	☐	☐	☐	☐	☐
H	If I did not guide or regulate my child's eating, he/she would eat too much of his/her favorite foods	☐	☐	☐	☐	☐

Q9:
Have you ever sought professional treatment for your child's weight?

Please select all that apply.

1. Yes, in a family-based program like the one described in this survey
2. Yes, through my child's doctor's office
3. Yes, through an organization like the YMCA
4. Yes, through my child's school or an afterschool program
5. Yes, through a program like Weight Watchers
6. Yes, other: [TEXTBOX]
7. No

© 2019 Wright DR et al. *JAMA Network Open.*

Q10:
Have you ever sought professional treatment for your own weight?

Please select all that apply.

1. Yes, through my doctor's office
2. Yes, through an organization like the YMCA, work, or my place of worship
3. Yes, through a program like Weight Watchers
4. Yes, through my gym
5. Yes, other: [TEXTBOX]
6. No

Q11:
How would you classify your child's race and ethnicity?

Please select all that apply.

1. White
2. Black or African American
3. Hispanic or Latino
4. Asian
5. Native Hawaiian
6. American Indian or Alaska Native
77. Don't Know
78. Some Other Group
79. Prefer not to answer

Q12.
How would you characterize the weight of your child:

1. Underweight
2. About the right weight
3. Slightly overweight
4. Overweight
5. Obese

Q13.
When your child is at home, how often are you responsible for feeding him/her?

1. Never
2. Seldom
3. Half of the time
4. Most of the time
5. Always

[SURVEY END]

© 2019 Wright DR et al. *JAMA Network Open.*
